# Faculty development for supervisors of medical student rural attachments in Zimbabwe

**DOI:** 10.4102/phcfm.v16i1.4641

**Published:** 2024-09-24

**Authors:** Fiona Makoni, Zandile Mafi, Sunanda Ray

**Affiliations:** 1 Department of Health Professions Education and Student Support, Faculty of Medicine and Health Sciences, University of Zimbabwe, Harare, Zimbabwe; 2 Department of Maternal and Child Health, Howard Mission Hospital, Chiweshe District, Zimbabwe

**Keywords:** rural attachment, distributed learning platforms, reflective writing, portfolios, faculty development, Zimbabwe

## Abstract

Reflective writing and keeping portfolios during rural hospital attachments has been shown to give medical students more confidence, better communication skills and clinical competence, thereby making them stronger adult learners. The role of supervisors as facilitators of learning during Community-based Education rural site visits is critical. The University of Zimbabwe Faculty of Medicine and Health Sciences established faculty development workshops to train supervisors to be better able to support students in becoming active learners and critical thinkers, giving them constructive feedback, and encouraging them to look for positive outcomes during their rural attachments. These educational skills were new to most supervisors, requiring a culture change from the usual didactic approaches. Educators had to learn about adult learning theories, the constructivist approach to knowledge creation, role-modelling and methods of assessment that were empowering for students. They were encouraged to form communities of practice through the faculty development process of training and assessment and to consider how to develop research and scholarship in documenting their experiences as facilitators and learning partners with their students and colleagues. These activities enhanced their professional identity formation as scholars, educators and facilitators. Their exposure to the functioning of rural hospitals, partly through narratives of the students’ reflective writing, enabled them to develop a greater appreciation of the potential of primary care and the district health services as the foundation of the Zimbabwe health system. Future benefits could include application of this training to other health professional programmes, as part of interprofessional education.

## Introduction

Contextual learning opportunities of clinical practice in primary and district hospital care can enrich medical students’ learning experiences, with improved clinical competencies, communication skills, relationships with colleagues and better appreciation of the health system.^1,2,3^ With increasing pressure to expand medical student intakes in order to meet health workforce goals, there have been moves towards distributed learning platforms that facilitate learning across various locations rather than being confined to tertiary academic institutions.^4,5^ By extending medical education to health facilities in rural and underserved settings, medical students potentially learn to apply theoretical knowledge and gain practical clinical experience in contexts of greater health need, while observing health-seeking behaviour and the influences of social determinants on health.

Experiential workplace-based learning of clinical practice, using problem-solving and reflective writing approaches, has been shown to ease students into being more active, self-directed learners and critical thinkers, rather than persisting as passive recipients of factual information.^6,7^ An essential factor for creating an effective workplace-based training environment is having clinical educators who are trained in relevant educational theory and skills, who are student-centred and support adult learning methods.^4^ In universities with large classes of medical students but few experienced educators who can facilitate this learning, students may not derive the anticipated benefits of their distributed learning experiences.^1,8^

## The context

Final-year medical students from the University of Zimbabwe Faculty of Medicine and Health Sciences (UZFMHS) have been involved in 4-week ‘field projects’ based at rural mission hospitals since 1968, with the objective of exposing them to patients’ illness journeys, disease patterns and rural health services.^9^ A revised undergraduate medical (MBChB) curriculum in 1987 endorsed the principle of training doctors to provide comprehensive healthcare including in district health services and to provide leadership in achieving equitable access to healthcare.^8,9^ The district and mission hospital experience has recently been amended to 4 weeks in year 5 and 2 weeks in the final year 6. There are currently 200 medical students per year who travel to 60 district or mission hospitals across the country, between 80 and 400 km away. The objectives of the rural hospital attachments are given in Table 1.

**TABLE 1:** Objectives of the Year 5 MBChB district/mission hospital attachments.^8,9^

**By the end of the attachment, each student will have studied and reflected in writing on the following:** Where and how people in Zimbabwe live and work What illnesses they experience What their perceived and actual health needs are What health care and health-related facilities are available How these facilities are organised at different levels to meet health needs, from communities and primary health care to district, provincial and tertiary levels of care
**Specific activities:** To assist with ward rounds, out-patients, operating theatre sessions and all other hospital activities, including with assessment and clinical management of individual patients To observe the referral of complex or emergency cases to provincial or tertiary hospitals To review the district health information system, comment on the quality of data provided and how it is used to improve quality of care To evaluate the services offered by the hospital laboratory and their utilisation, with a specific focus on the range of tests available, the interpretation and application of the results To conduct a home visit to at least one in-patient clerked by one of the students and assess: (1) the social background of the patient; (2) the implications of the illness; and (3) the patient’s and the family’s beliefs about the illness

*Source:* Rural attachment office, Faculty of Medicine & Health Sciences, UZ

MBChB, Bachelor of Medicine and Bachelor of Surgery Degree.

During rural hospital attachments, day-to-day supervision of medical student activities is conducted by medical officers or senior nurses who have little formal training in medical education and tend to replicate the teaching styles they received as students. Academic faculty from the university visit once during each attachment, and include non-clinicians (laboratory scientists, lecturers in biomedical sciences, statisticians for example) because of the numbers of students spread across several sites.^8^ Hospital specialists rarely visit students at these rural sites. More clinicians supervise the 2-week Year 6 rural attachment, which has a stronger clinical focus.

The medical students recently learnt to develop portfolios with reflective narratives of clinical cases they were involved with at rural hospitals. Constructivist learning theory posits that learners actively construct their understanding and knowledge through integrating new experiences into prior knowledge, with reflection and engagement.^10^ Reflective writing facilitates this learning, enabling students to consolidate their insights as they attempt to process the complexity of situations they face, developing clinical and professional skills and enhancing professional identity formation as health professionals.^2,11^ They learnt the Driscoll model of reflection, that of description (noticing), sense-making (processing) and future action, helping them to critically analyse their experiences and plan for future improvement.^12^ Non-clinical supervisors, though confident of the skills of their discipline, felt less so when faced with facilitating and assessing journal entries written by medical students that focused on generalist clinical scenarios, especially in relation to reflections that are exploratory, emotion centred and experiential.

Good facilitation is essential for students to express themselves without fear of judgement or censure.^13,14^ In response to this challenge, the Department of Health Professions Education (DHPE), established in 2014 at UZFMHS, created a set of faculty development (FD) workshops to provide educational skills for facilitators, which are described here. This paper describes an intervention to support academic faculty as facilitators of the students’ rural attachment experience, to become more learner centred as educators, to enable medical students to get higher value from their clinical exposure in rural hospitals and to develop as critical thinkers and self-directed learners.

## The innovation

Two FD workshops were conducted to enable supervisors to effectively lead, support and assess learners, to better meet the MBChB rural attachment learning objectives. The objectives of the workshops were to ensure that supervisors for the MBChB rural attachment:

- Engage with the principles and goals of rural attachment, including its role in meaningful learning experiences in rural settings that align with educational objectives and competencies.
- Explore the role of reflective writing in effective lifelong learning practices and providing constructive feedback to learners.
- Acquire facilitation skills, encouraging students to become active learners by asking questions of themselves, their colleagues and health staff without being perceived as being challenging or confrontational.
- Develop scholarship and professional identity formation as educators of health professional students.

The first, a two-day workshop, focused on providing the underpinning theoretical principles of adult learning and reflective writing, distributed learning platforms, the learning objectives of the rural attachment and giving verbal feedback to the students on their portfolios. The importance of reviewing the students’ reflections as a group so they could learn from each other, ask questions and give each other answers was emphasised as the focal point of the faculty supervisory visits. The importance of constructive feedback as a driver of learning, intrinsic motivation and improved performance was explored. Giving feedback was explained using ‘Pendleton Rules’ where learners first reflect on what they felt went well in their performance, followed by the facilitator reinforcing these positives; learners then suggest improvements and receive guidance from the facilitator on how to achieve these.^15^ Examples of giving feedback were demonstrated showing that it should be specific and goal oriented, descriptive, non-judgemental, based on observed behaviours and delivered in a sensitive, timely and consistent manner. It should also be manageable, actionable and framed as a dialogue.^15^ Providing a safe environment for students to receive and act on feedback, with the goal of improving their performance and self-efficacy, was a crucial skill for facilitators to become more learner centred. Supervisors were encouraged to look for narratives with positive outcomes (appreciative inquiry)^16^ for the students from their rural experiences, as reinforcement of positive practices and to identify actions and role models to emulate for future practice.^7^

The workshop sessions were interspersed with practical interactive exercises for faculty to practise new skills with this approach and act as facilitators rather than teachers. The change from didactic teaching, the comfort zone of most medical faculty, to be supportive of students as adult learners, requires a significant culture change. Reflective writing promotes problem solving, critical thinking and student oriented rather than subject-oriented learning, which enhances learning for students as they become more confident of their interpretations of their experiences. It could also be transformative for supervisors through their interactions with students as adult learners, as they reconsider their own beliefs, values and assumptions about themselves as educators. Faculty from diverse disciplines explored how to discuss the students’ narratives from the perspective of their own knowledge base but in the context of the students’ learning. For example, a laboratory scientist could discuss a case of malaria from the perspective of the students’ enquiry of laboratory tests conducted.

The second FD workshop focused on principles of assessment in HPE, focusing on how to evaluate the portfolios as the summative assessment of each student’s rural attachment. During this workshop, faculty brought the student portfolios they were marking from the site visits they made and discussed in groups, examples of learning demonstrated by the students in the portfolios. This exercise helped to guide the group in application of the marking rubric and to agree on the scores given (see Table 2) in alignment with the rural attachment objectives. Subsequently, the marking would be conducted independently by the supervisors. The focus was not on accuracy of factual information, but on how learners linked past knowledge with current dilemmas to suggest how to apply their learning to future activities.

**TABLE 2:** Reflective learning marking rubric.

Marks	Criteria
9-10 (A)	Students form opinions based on the course and experience plus other sources, with evidence of effectiveness and linkages between reflections and course material.
7-8 (B)	Students evaluate material using concepts from the course and merge with experience. Analysis of factors from experience that contributed to learning and educational development.
5-6 (C)	Students are actively questioning and considering multiple perspectives and opinions that have some analysis. Discussion of learning encounters is well supported with examples, challenges, techniques and lessons learned.
1-4 (D)	Students accept things at face value, giving superficial factual accounts of events without demonstrating learning or educational development.

*Source:* Developed through Faculty Development Workshop Department of Health Professions Education, University of Zimbabwe

Faculty were encouraged to proactively plan their site visits, to interact with their student group before they travelled to the attachments, to familiarise themselves with the learning objectives, to clarify expectations and to stimulate the students’ active participation. Examples of scholarship in FD from other HPE centres were shared at the workshop, which prompted faculty to consider documenting their own experiences, to develop stronger professional identities as educators and scholars. Through this collaborative learning partnership between educators and students, a richer appreciation of the pivotal role of district health services and primary care in the educational system emerged, rather than being judged as peripheral and mediocre. In future, the effectiveness of the FD programme in achieving its objectives will be evaluated using focus group discussions to assess faculty and student satisfaction with their rural attachment experience and continued participation in further FD events.

## Discussion

A well-facilitated supervisory process is critical for distributed learning platforms to be effective for training medical students to become self-aware, life-long active learners.^7^ The value that was gained through the FD workshops was an appreciation that all supervising faculty can contribute towards students’ learning, whether they are clinicians or not. They developed into communities of practice, which are networks of educators with shared knowledge, values, expertise and a vision of how to collectively achieve their purpose and support each other in their professional identity formation as educators and scholars.^7,13,17^

A review of the rural attachment programme in 2015 recommended that the ‘field attachment’ be converted into a formal Community-based Education (CBE) programme with improved supervision and clearer competency-based learning objectives aligned to the medical curriculum. Such CBE programmes draw heavily on knowledge and skills usually associated with family medicine (FM): continuity of care that is holistic and comprehensive. There are, however, currently only two FM-trained specialists in Zimbabwe and both hold faculty positions at the university. In future, CBE educators who are FM specialists with training in medical education will lead the inputs of this part of the MBChB curriculum. Following the initiation of the postgraduate MMed FM in 2020, UZ has incorporated undergraduate FM into year 5 of the MBChB programme, starting September 2024, which will absorb the 4-week rural attachment as a CBE rotation. Improvements to the supervisory framework will include continued FD for academic supervisors and training for non-academic supervisors at the host rural hospitals, for instance in giving constructive feedback, supporting reflective writing and self-directed learning. Lessons learnt from the MBChB CBE and FD programme could be applied to other health professional (nursing, pharmacy, rehabilitation, etc.) CBE programmes especially if they are incorporated as interprofessional education and contribute to team-building and health system strengthening.

## Conclusion

This short report highlights the challenges faced by universities in trying to develop community-based medical education programmes in the context of increasing medical student intakes, taught by academic supervisors who tend to be tertiary hospital centred because of their backgrounds and expertise. Faculty supervising medical students during their rural attachments, clinicians and non-clinicians alike tend to be teacher and subject centred and are not familiar with methods of teaching medical students to be self-directed, critical thinking, active learners. The innovation of FD through the DHPE aims to enable these educators to support medical students develop as adult learners through the use of distributed learning platforms that include district and mission hospitals in rural areas. By supporting students to develop experiential adult learning techniques through reflective writing and peer discussions, academic educators gain expertise themselves in providing constructive feedback, supervision and assessment of reflective writing in portfolios and promoting self-directed learning.

The authors wish to thank the un-named reviewer(s) whose comments helped to strengthen the paper.
